# Supplementary material for: Glioma synapses recruit mechanisms of adaptive plasticity
Source: Nature. 2023 Nov 1;623(7986):366–74. doi: 10.1038/s41586-023-06678-1 (PMC10632140; doi:10.1038/s41586-023-06678-1)
Supplement: Supplementary file 1 — Original western blots. [file 41586_2023_6678_MOESM1_ESM.docx]

Ladders:

L1) WesternSure Pre-stained Chemiluminescent Protein Ladder (#926-98000, LICOR Biosciences)

L2) Precision Plus Protein Dual Color Standards (#1610374, Bio-rad)

**Figure 3**

**b) AMPA Receptor 4 - GluA4 (#8070, Cell Signaling Technology)**

Total Protein

Cell Surface

+

5m

-

BDNF

+

30m

+

15m

-

+

5m

+

15m

+

30m

kDa


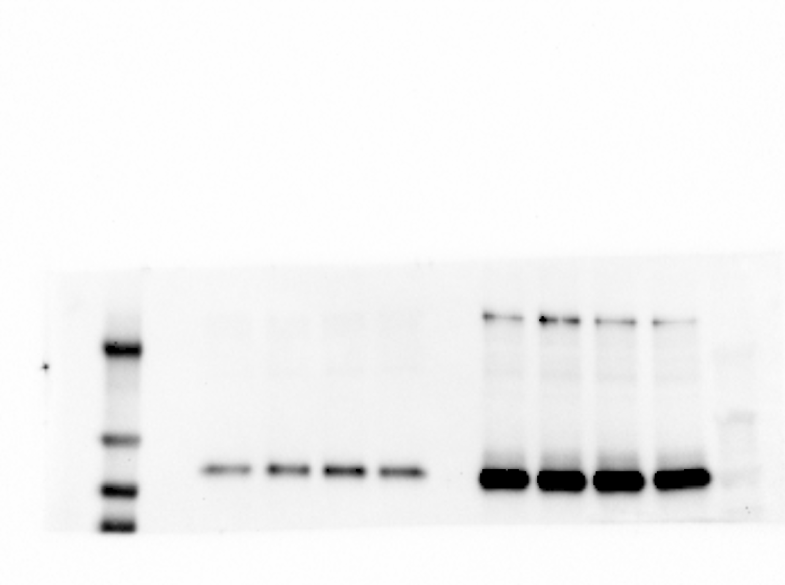


250

125

90

L2

70

L1

**GAPDH (#5174, Cell Signaling Technology)** (Run on the same gel)

Cell Surface

+

5m

-

Total Protein

+

30m

+

15m

BDNF

-

+

5m

+

15m

+

30m

kDa


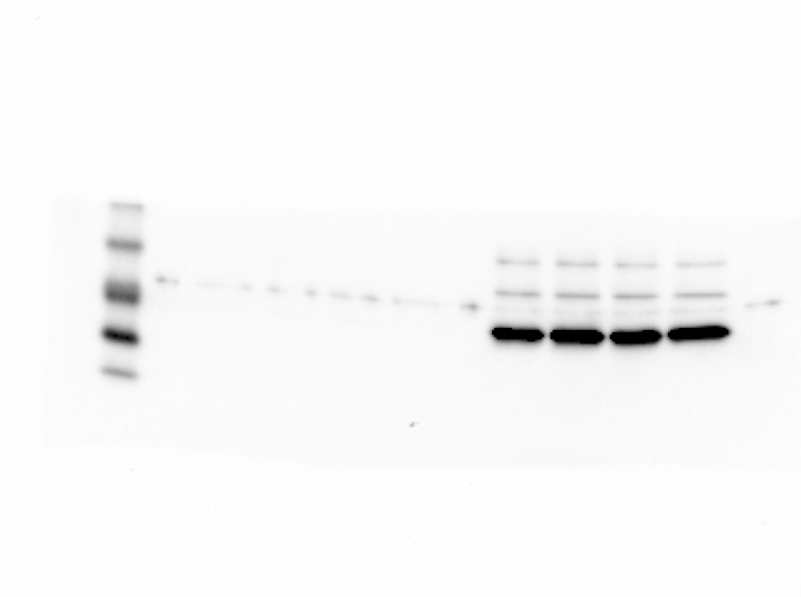


50

38

25

15

L2

L1

**d) AMPA Receptor 3 – GluA3 GAPDH (#5174, Cell Signaling Technology)**

**(#4676, Cell Signaling Technology)** (Run on the same gel)

BDNF

-

Cell Surface

-

Total Protein

Total Protein

-

Cell Surface

-

kDa

+

+

+

+

BDNF


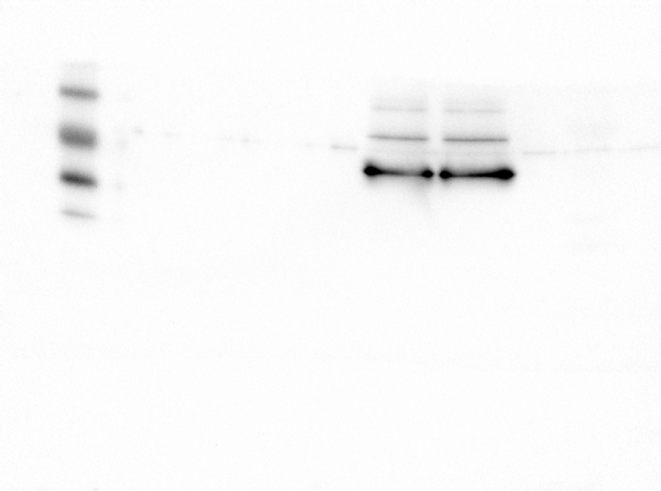

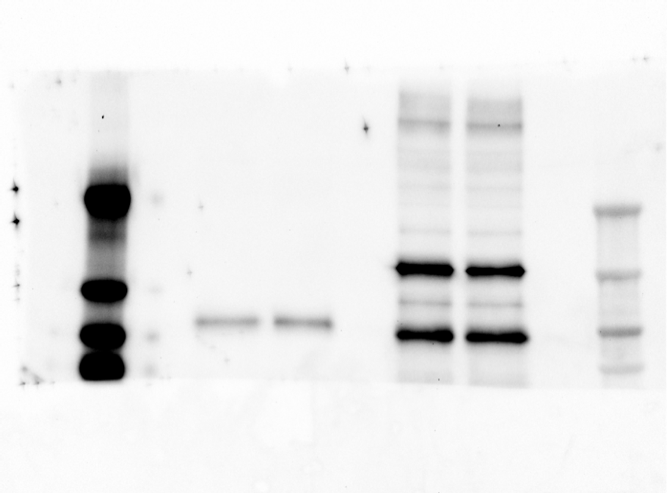


L2

L1

L2

L1

15

38

25

50

250

125

90

70

100

75

150

250

kDa

kDa

**f) AMPA Receptor 4 - GluA4 (#8070, Cell Signaling Technology)**

250

-

+

Total Protein

-

Cell Surface


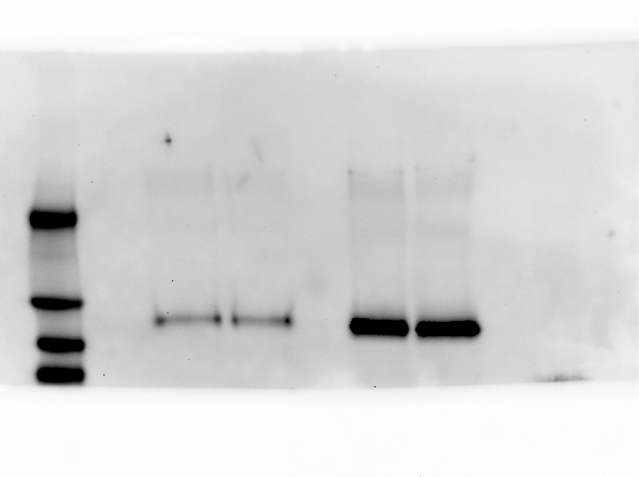


125

NLGN3

+

250

125

90

70

L1

**GAPDH (#5174, Cell Signaling Technology)** (Run on the same gel)

NLGN3

-

+

Cell Surface

-

+

Total Protein


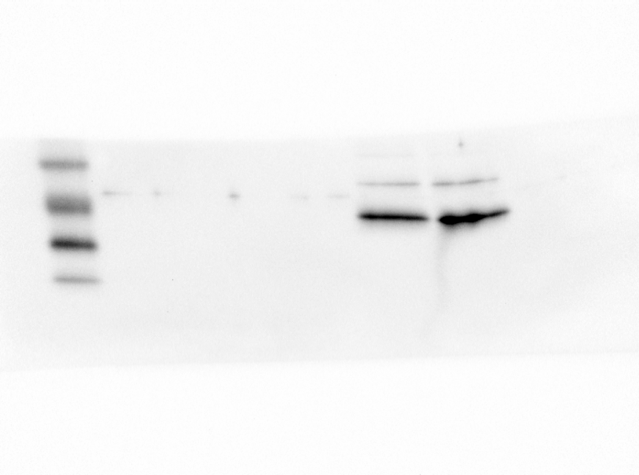


50

15

25

38

L1

**Extended Data Figure 1**

**g) TrkB (#4606, Cell Signaling Technology)** MW 140 kDa Full Length, ~90 kDa Truncated-SHC, Truncated T1.

pcGBM2

DIPGXIII

DIPGVI


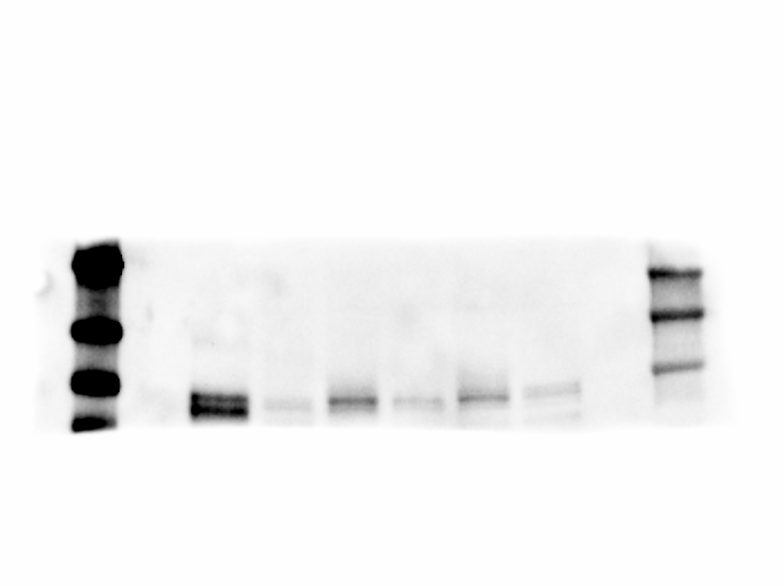


L2

L1

WT KO

WT KO

WT KO

70

125

250

90

250

150

100

**b-actin (#4970, Cell Signaling Technology)** (Run on the same gel).

L2

L1

WT KO

pcGBM2

DIPGXIII

DIPGVI

WT KO

WT KO

38

50

25


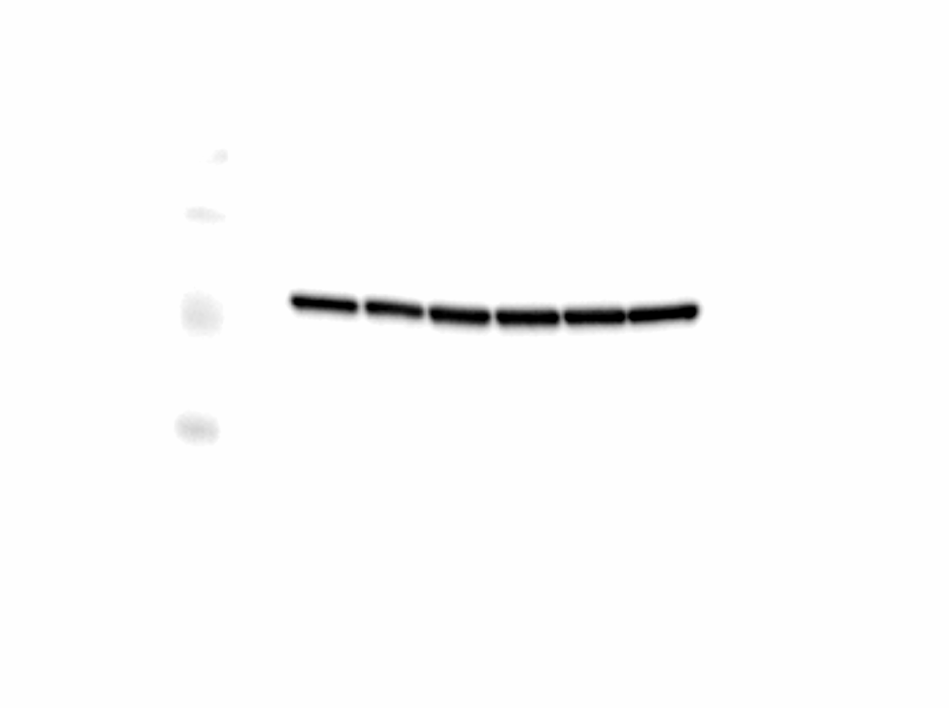


**Extended Data Figure 2**

1. **pTrkB (pTyr515) – (NB100-92656, Novus Biologicals)** MW 140 kDa Full Length, ~90 kDa Truncated-SHC, Truncated T1.


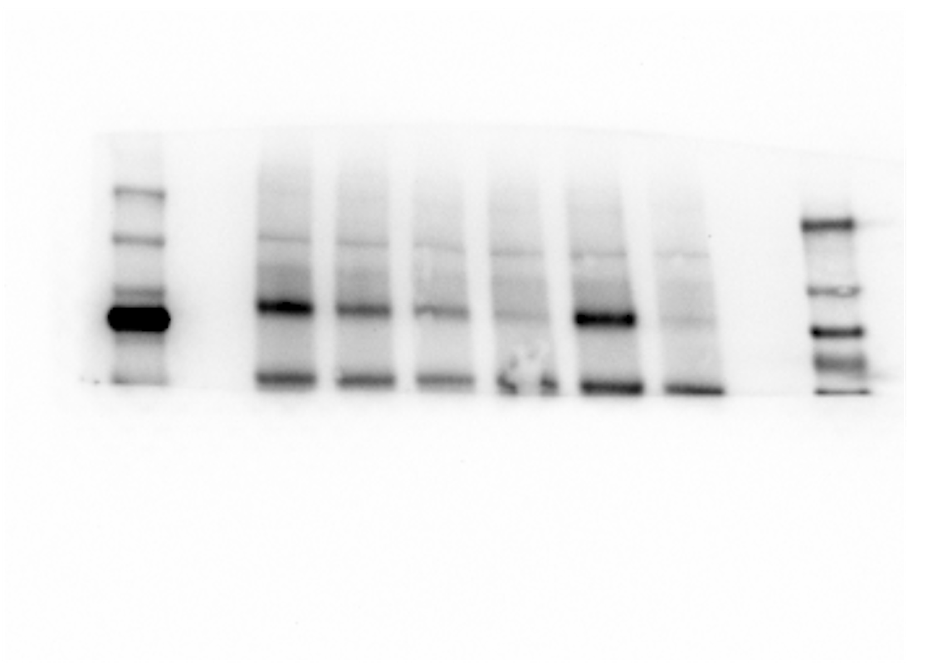


2h

4h

30m

Veh Ent

Veh Ent

Veh Ent

75

250

150

100

70

90

125

250

L2

L1

**TrkB (#4606, Cell Signaling Technology)** MW 140 kDa Full Length, ~90 kDa Truncated-SHC, Truncated T1. Same sample run on separate gel to phospho antibody.

Left, blot used in Figure (ladders covered to expose blots), right is the same blot without ladders covered.

30m

2h

4h

Veh Ent

Veh Ent

Veh Ent


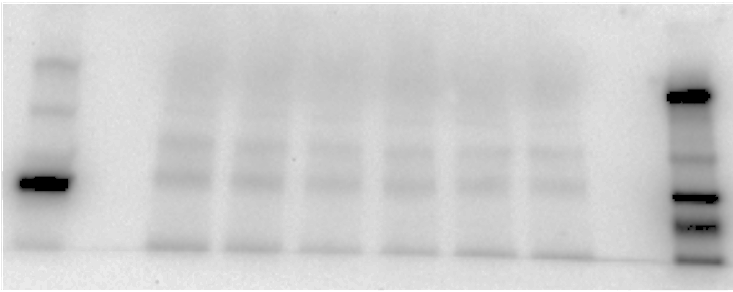


250

125

70

90

Veh Ent

Veh Ent

Veh Ent

30m

2h

4h


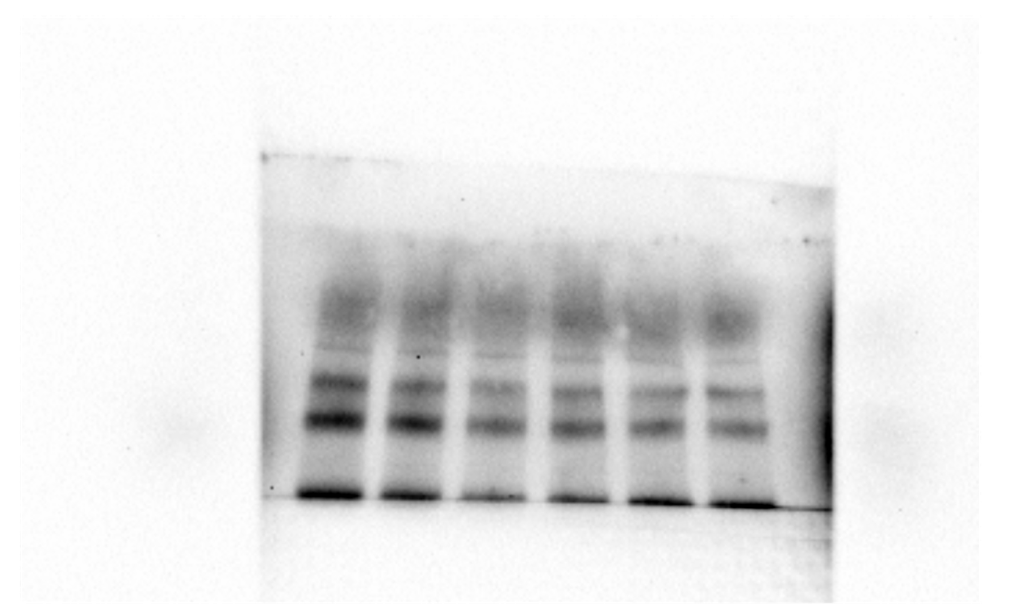


L1

L2

**Phospho-p44/42 MAPK (ERK1/2) (Thr202/Tyr204) – (#4370, Cell Signaling Technology)**

Veh Ent

30m

Veh Ent

4h

2h

L2


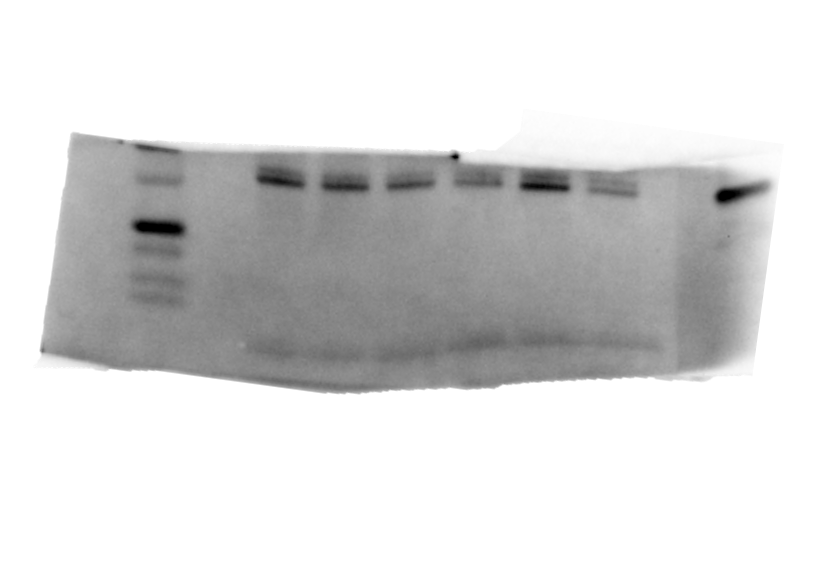


25

50

37

Veh Ent

**p44/42 MAPK (Erk1/2) – (#9102, Cell Signaling Technology)**

(left ladder not in the image frame). Same sample run on separate gel to phospho antibody.

Veh Ent

Veh Ent

30m

2h

4h


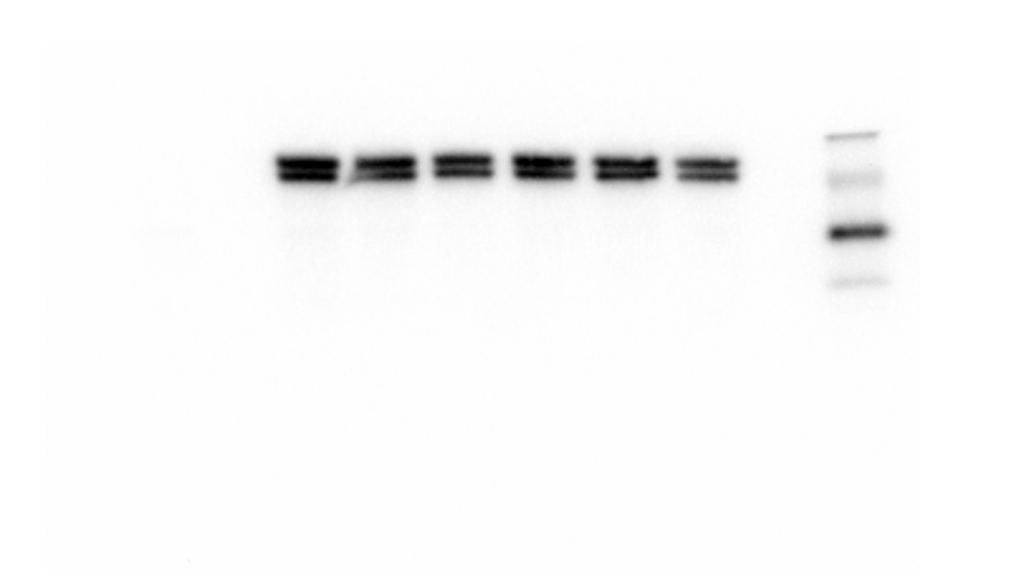


L1

38

50

25

Veh Ent

**b-actin (#4970, Cell Signaling Technology)**

Same gel as phospho TrkB.

Veh Ent

Veh Ent

30m

2h

4h

Veh Ent


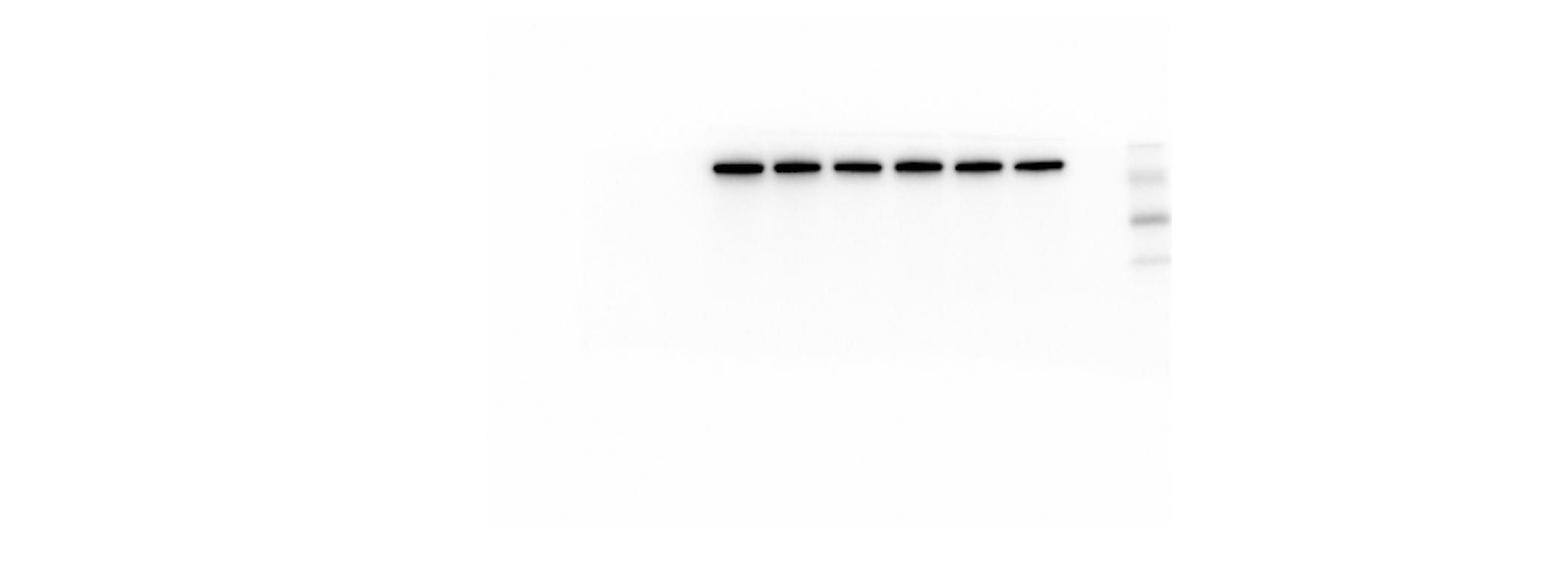


L1

50

25

38

**Extended Data Figure 8**

1. *These are the same experimental samples/lysates used in Ext Data Fig 8e.

** The same protein lysate sample (+load and SRA) was used for all blots.

**Phospho-p44/42 MAPK (ERK1/2) (Thr202/Tyr204) – (#4370, Cell Signaling Technology)**

BDNF

+

15m

+

5m

-

+

30m

25

38

50

L1


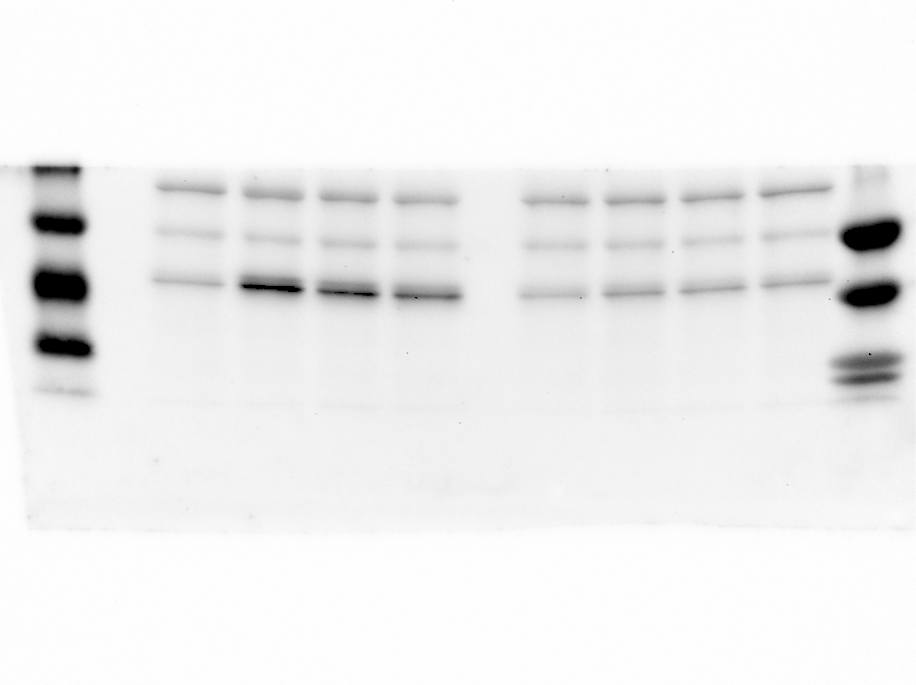


**p44/42 MAPK (Erk1/2) – (#9102, Cell Signaling Technology)**

Same sample run on separate gel to phospho antibody.

+

30m

+

15m

+

5m

-

BDNF

L1


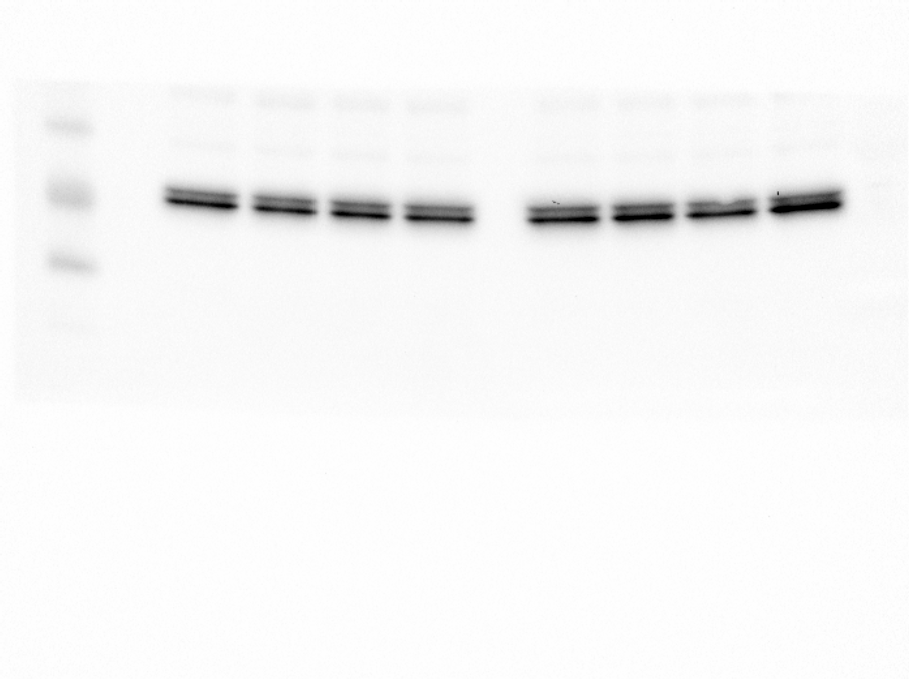


25

38

50

**Phospho-AKT (Ser473) – (#4060, Cell Signaling Technology)**

+

30m

+

15m

+

5m

-

BDNF


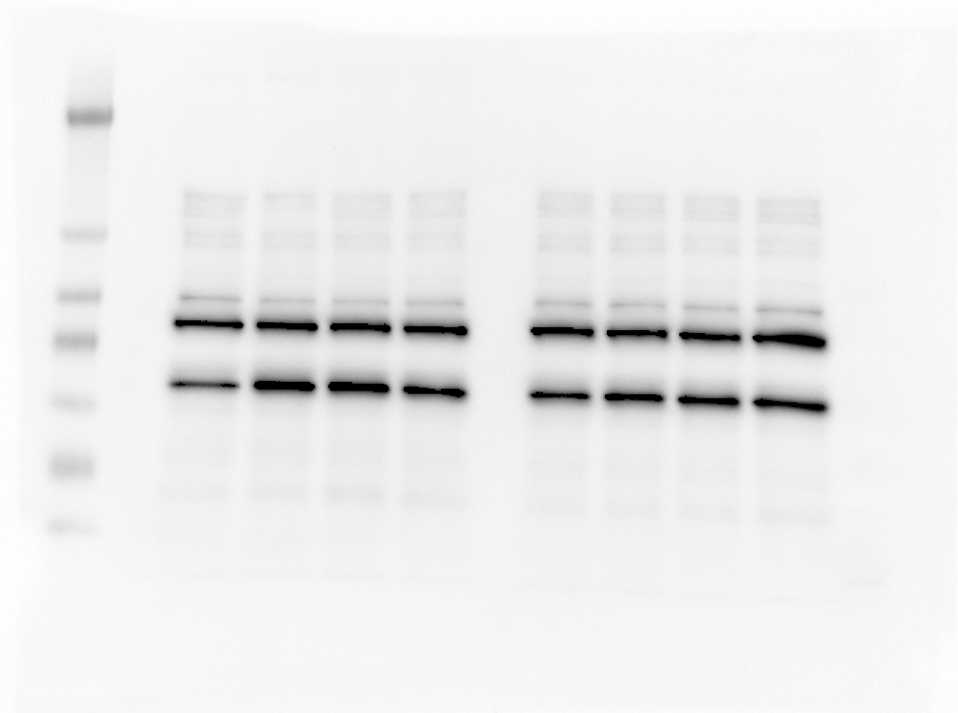


90

70

25

50

38

L1

**AKT antibody (#9272, Cell Signaling Technology)**

*First blot exhibited sample depletion, membrane was striped and re-probed with reduced antibody concentration. Striped blot lost chemiluminescent ladder. Right, initial blot, left re-probed blot used in Figure. *note presence of lower non-specific bands in both blots.

Same sample run on separate gel to phospho antibody.


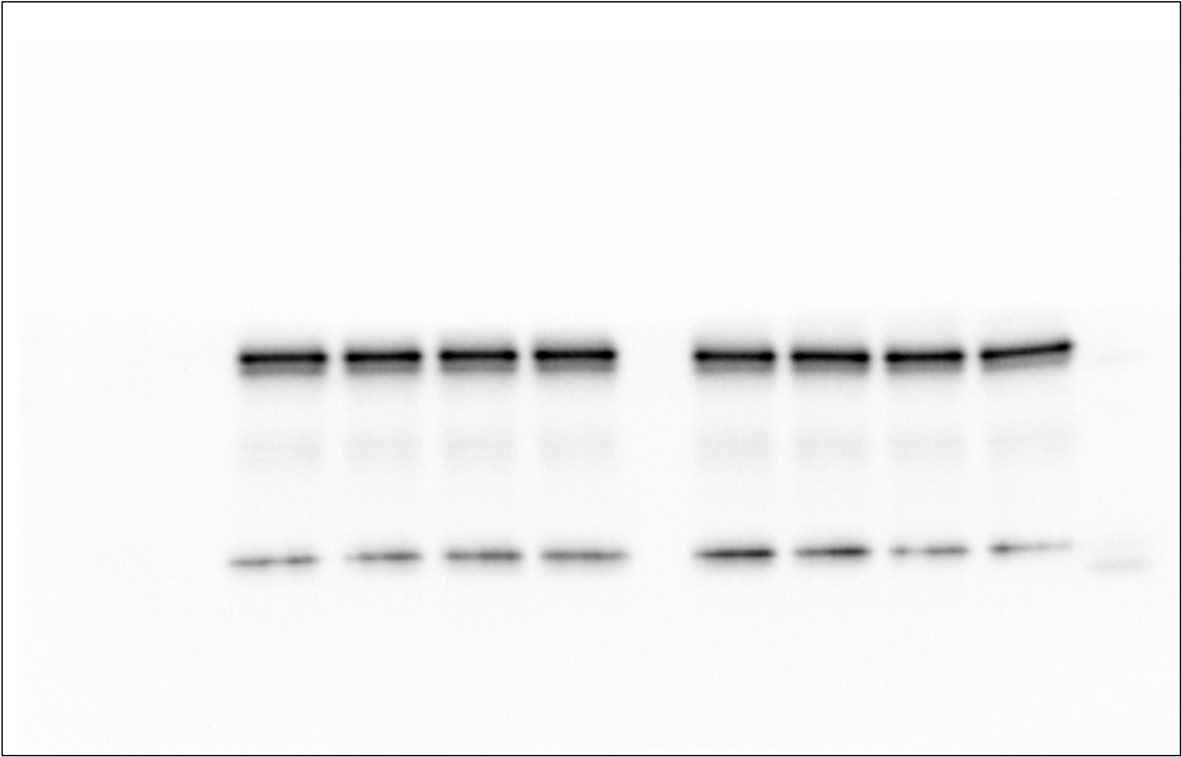

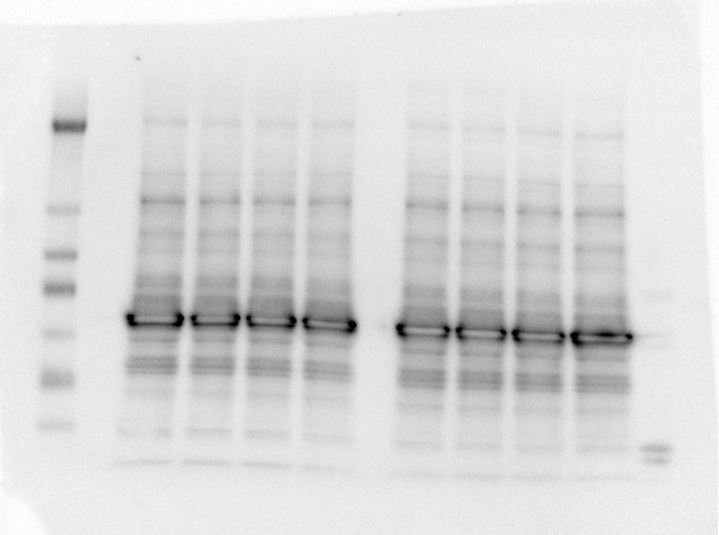


*

*

BDNF

-

+

15m

+

30m

+

5m

BDNF

-

+

5m

+

15m

+

30m

125

38

50

70

90

L1

**phospho-CAMKII (Thr286) – (#12716, Cell Signaling Technology)**

MW 50 kDa CAMKII-α (analyzed), 60 kDa CAMKII-β

+

5m

+

15m

+

30m

BDNF

-

L1


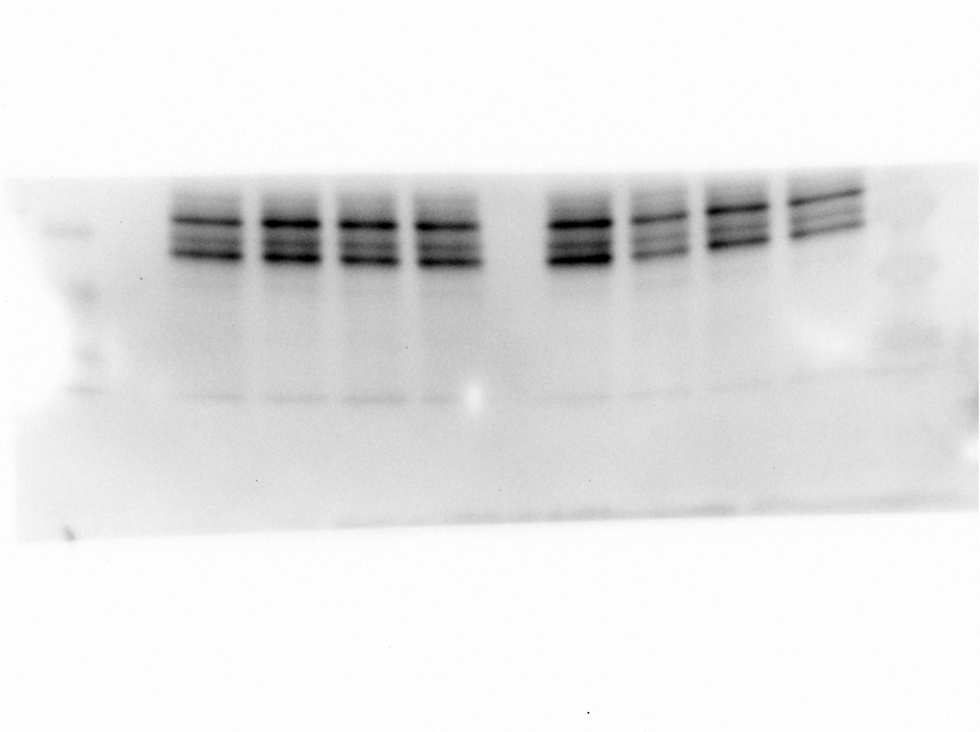


50

38

25

**CAMKII (#4436, Cell Signaling Technology)**

MW 50 kDa CAMKII-α (analyzed), 60 kDa CAMKII-β
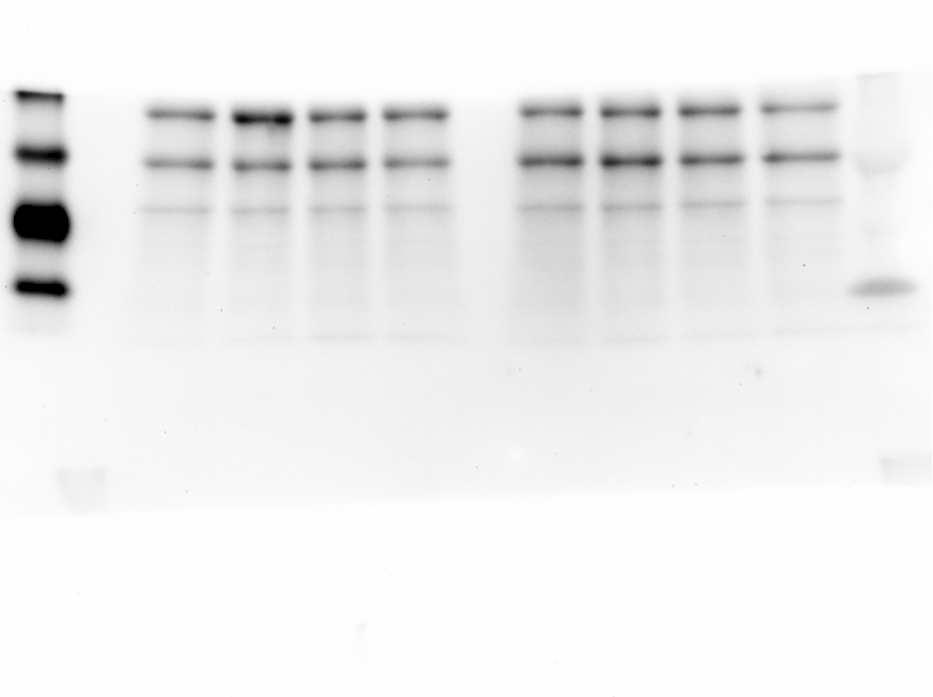
. Same sample run on separate gel to phospho antibody.

+

30m

+

15m

+

5m

-

BDNF

L1

50

70

38

25

**b-actin (#4970, Cell Signaling Technology)**

+

15m

+

5m

-

+

30m


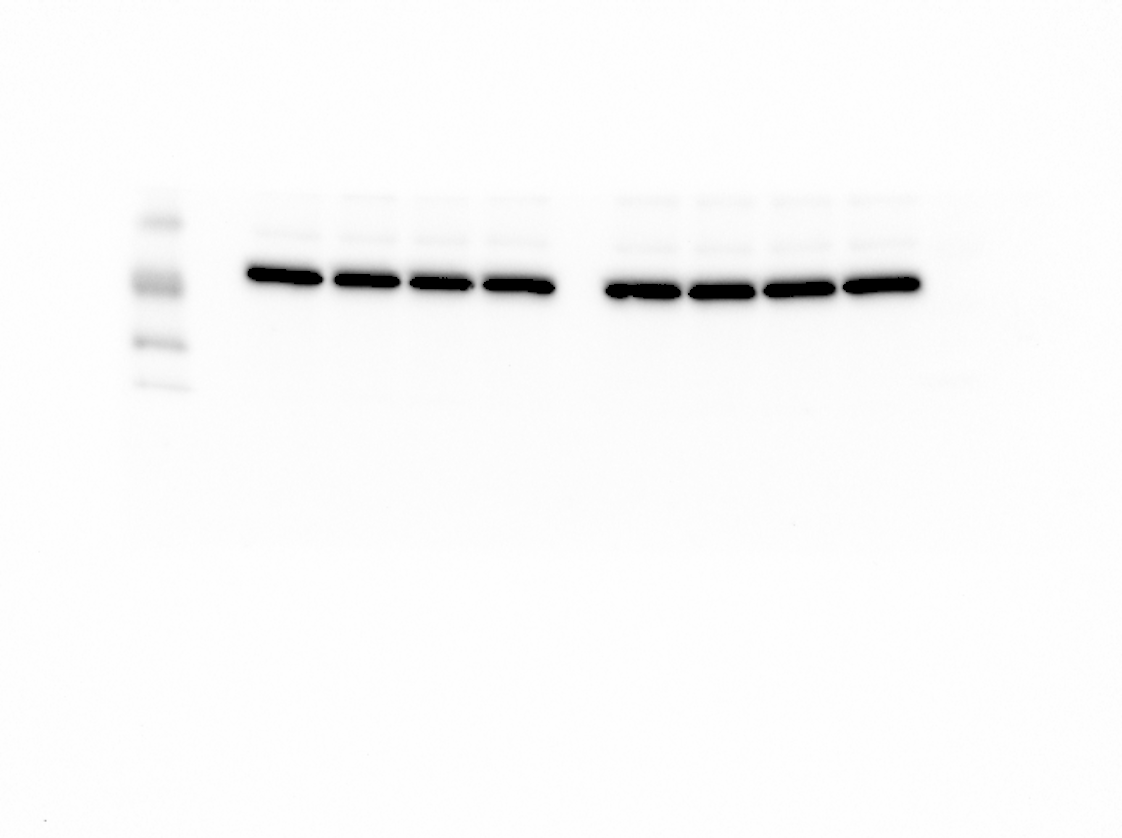


BDNF

L1

38

25

50

**e)** *These are the same experimental samples/lysates used in Ext Data Fig 8a.

** The same protein lysate sample (+load and SRA) was used for all blots.

**Phospho-GluA4 (Ser862) – (PA5-36807, Invitrogen)**


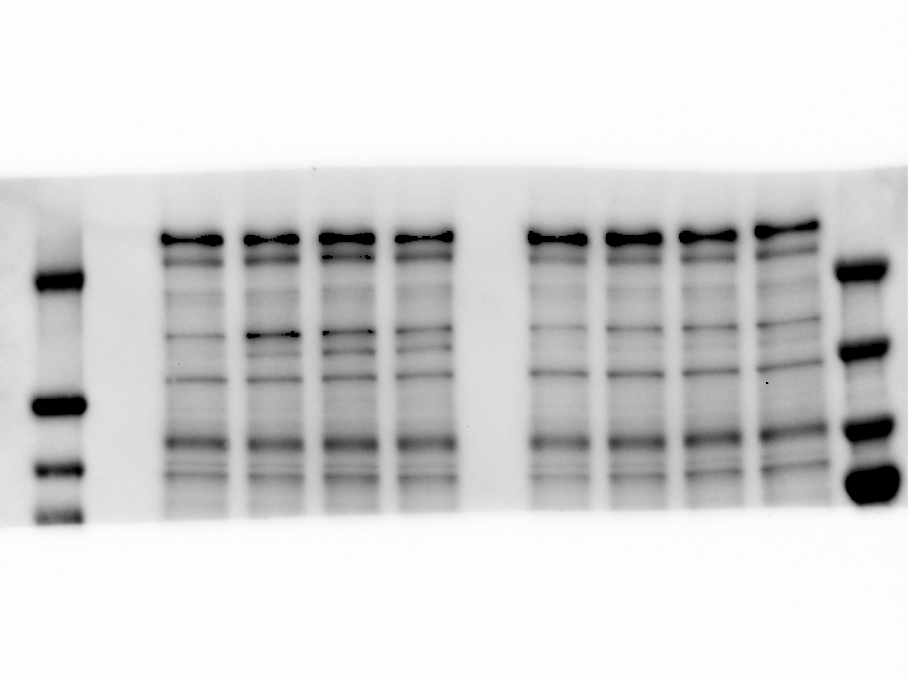


250

125

90

BDNF

-

+

5m

+

15m

+

30m

L1

**AMPA Receptor 4 - GluA4 (#8070, Cell Signaling Technology)**

Same sample run on separate gel to phospho antibody.

BDNF

-

+

5m

+

15m

+

30m


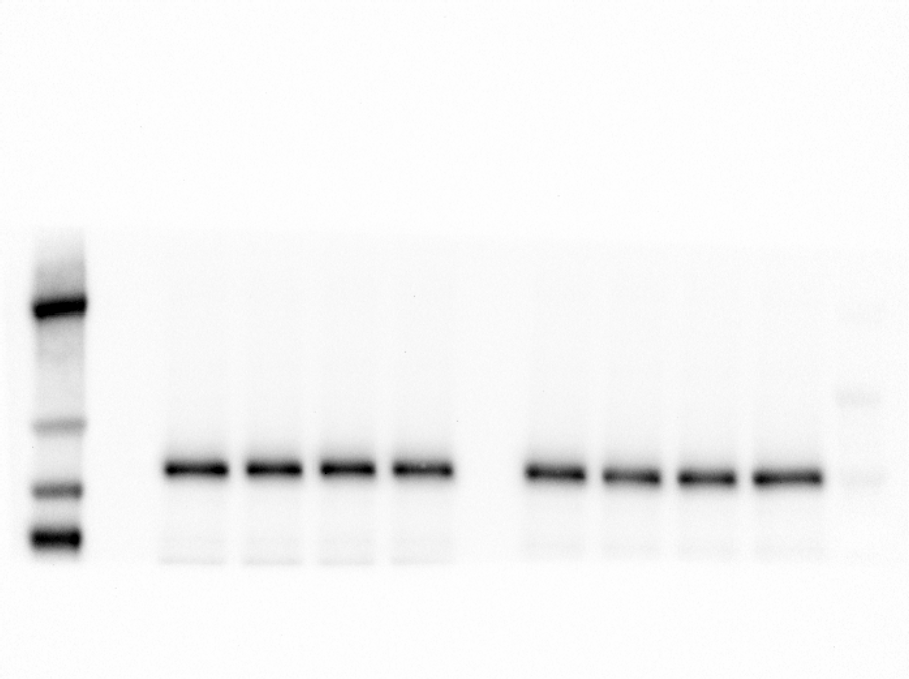


250

125

90

**b-actin (#4970, Cell Signaling Technology**)

+

30m

+

15m

+

5m

-

BDNF


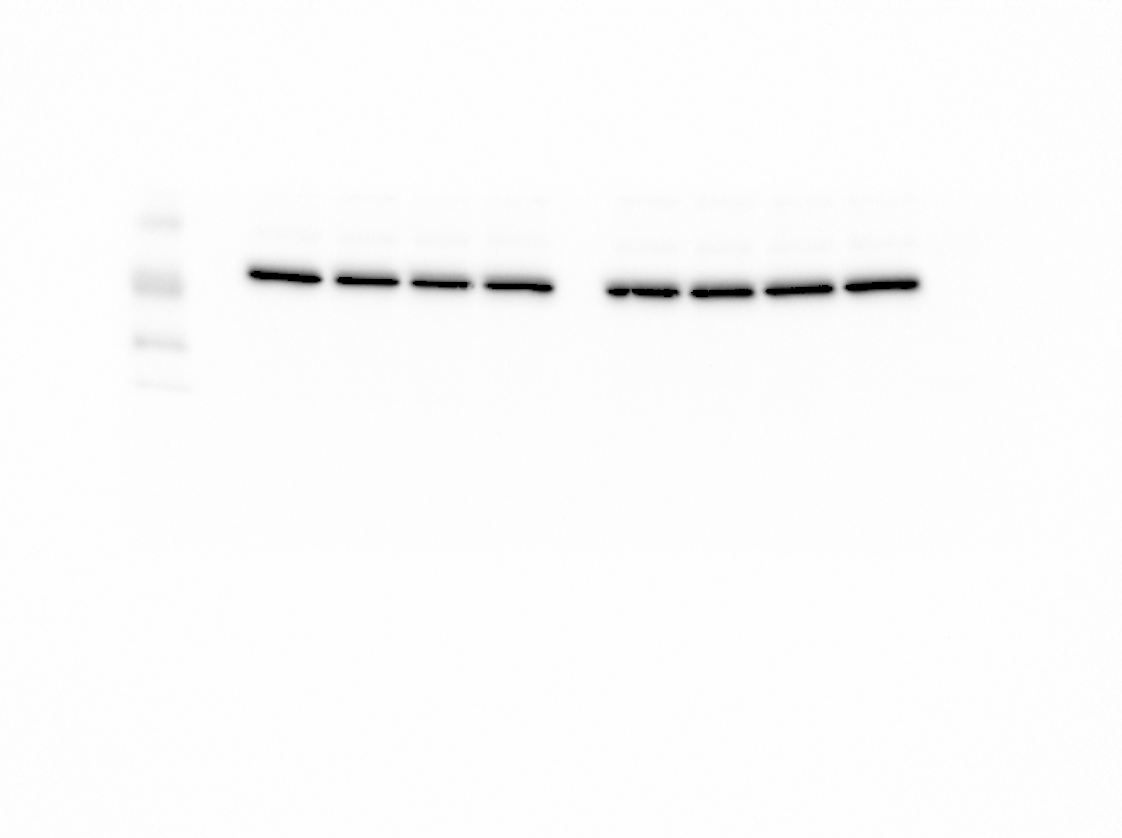


25

38

50

L1

**g) Phospho-GluA4 (Ser862) – (PA5-36807, Invitrogen)**

+

+

-

BDNF


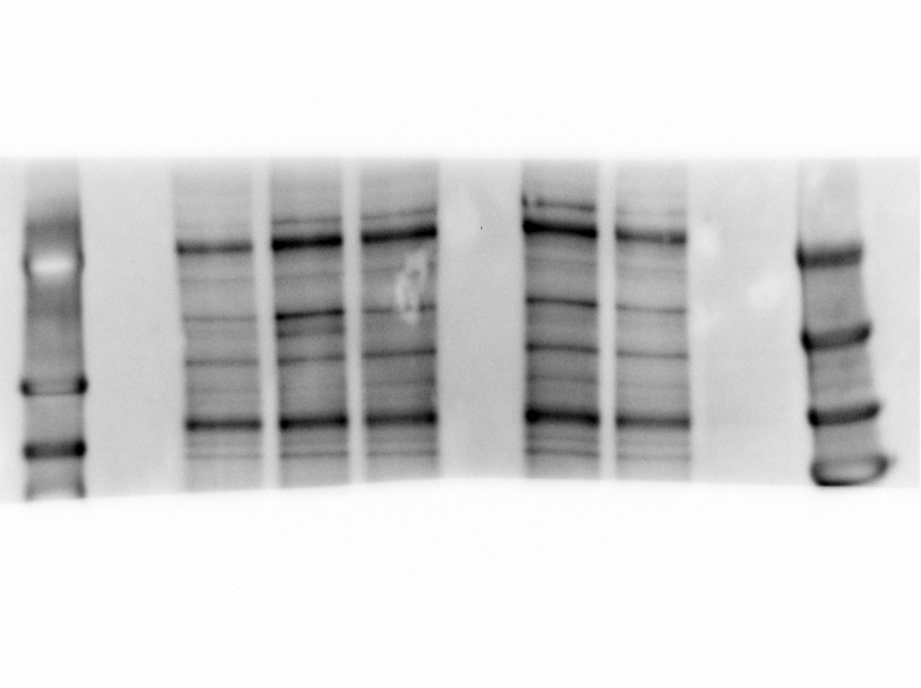


+

-

-

Entrectinib

90

250

125

L1

**AMPA Receptor 4 - GluA4 (#8070, Cell Signaling Technology)**

Same sample run on separate gel to phospho antibody.

+

-

-

Entrectinib

+

+

-

BDNF


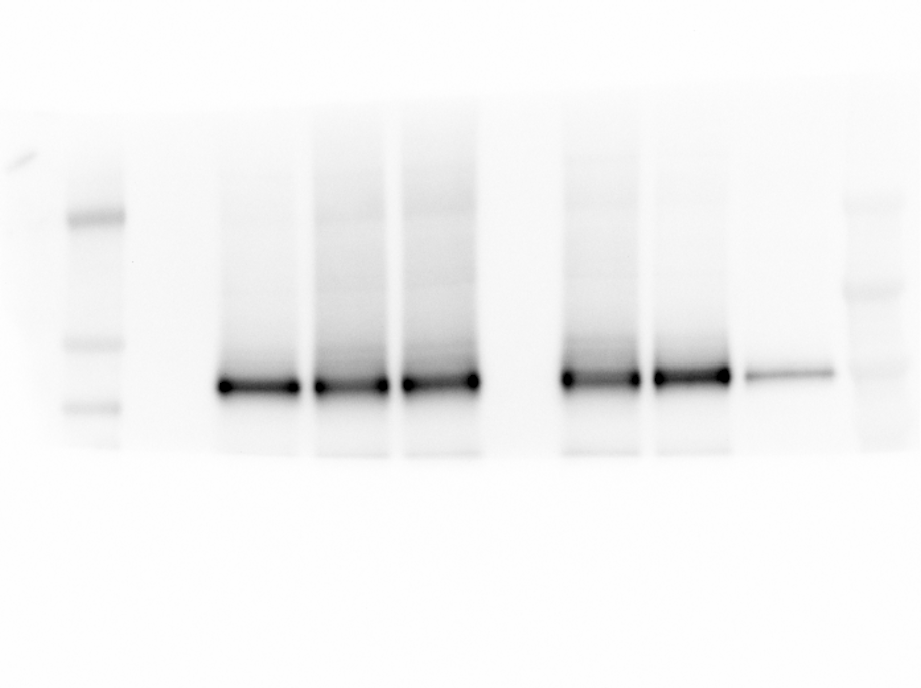


125

90

250

L1

**b-actin (#4970, Cell Signaling Technology**)

-

-

Entrectinib

+

+

-

BDNF


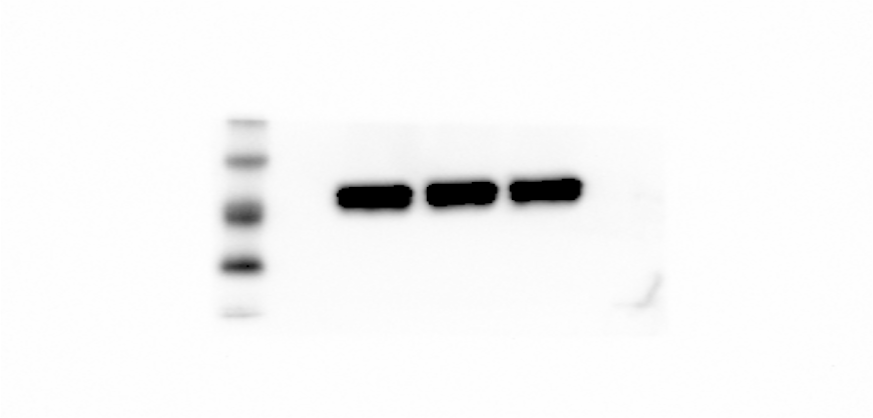


+

70

L1

25

38

50

Supplementary Figure 1: All full western blots. Molecular weight ladder on left (lane 1, L1) of all western blots. The bands of interest highlighted in red.
