# Supplementary material for: Glioma synapses recruit mechanisms of adaptive plasticity
Source: Nature. 2023 Nov 1;623(7986):366–74. doi: 10.1038/s41586-023-06678-1 (PMC10632140; doi:10.1038/s41586-023-06678-1)
Supplement: Supplementary file 2 — Reporting Summary [file 41586_2023_6678_MOESM2_ESM.pdf]

nature portfolio

Corresponding author(s): Michelle Monje

Last updated by author(s): 9/19/2023

Reporting Summary

Nature Portfolio wishes to improve the reproducibility of the work that we publish. This form provides structure for consistency and transparency in reporting. For further information on Nature Portfolio policies, see our Editorial Policies and the Editorial Policy Checklist.

Statistics

For all statistical analyses, confirm that the following items are present in the figure legend, table legend, main text, or Methods section.

n/a	Confirmed	
☐	☒	The exact sample size (*n*) for each experimental group/condition, given as a discrete number and unit of measurement
☐	☒	A statement on whether measurements were taken from distinct samples or whether the same sample was measured repeatedly
☐	☒	The statistical test(s) used AND whether they are one- or two-sided<br>*Only common tests should be described solely by name; describe more complex techniques in the Methods section.*
☐	☒	A description of all covariates tested
☐	☒	A description of any assumptions or corrections, such as tests of normality and adjustment for multiple comparisons
☐	☒	A full description of the statistical parameters including central tendency (e.g. means) or other basic estimates (e.g. regression coefficient) AND variation (e.g. standard deviation) or associated estimates of uncertainty (e.g. confidence intervals)
☐	☒	For null hypothesis testing, the test statistic (e.g. *F*, *t*, *r*) with confidence intervals, effect sizes, degrees of freedom and *P* value noted<br>*Give P values as exact values whenever suitable.*
☒	☐	For Bayesian analysis, information on the choice of priors and Markov chain Monte Carlo settings
☒	☐	For hierarchical and complex designs, identification of the appropriate level for tests and full reporting of outcomes
☐	☒	Estimates of effect sizes (e.g. Cohen's *d*, Pearson's *r*), indicating how they were calculated

Our web collection on statistics for biologists contains articles on many of the points above.

Software and code

Policy information about availability of computer code

Data collection	Electrophysiology data were collected with a MultiClamp 700B amplifier (Molecular Devices) and digitized at 10 kHz with InstruTECH LIH 8+8 data acquisition device (HEKA) and analyzed using AxoGraph X and IgorPro v.8. Calcium imaging data was either collected on a microscope equipped with DIC optics (Olympus BX51WI) and FlyCapture 2.13 release 61 software, or a Prairie Ultima XY upright two-photon microscope equipped with an Olympus LUM Plan FI W/IR-2 40x water immersion objective and Prairie View 5.6 software. Confocal images were acquired on either a Zeiss Airyscan1 LSM800 or Zeiss Airyscan2 LSM980 using Zen 2011 v8.1.
Data analysis	Statistical tests were conducted using GraphPad Prism v9.1.0 software for most analysis. For analysis of previously published scRNAseq data, R studio 1.4.1106-5 was used, with packages clusterProfiler v3.18.1, Kallisto v.0.46.1, Tophat2 v2.0.1.3, featurecounts v2.0.3 and DESeq2 v1.36.0 and EnhancedVolcano v1.14.0. Calcium imaging and pHluorin analyses were performed using ImageJ v.2.1.0/1.53c with standard ROI intensity plugin.

For manuscripts utilizing custom algorithms or software that are central to the research but not yet described in published literature, software must be made available to editors and reviewers. We strongly encourage code deposition in a community repository (e.g. GitHub). See the Nature Portfolio guidelines for submitting code & software for further information.

Data

Policy information about availability of data

All manuscripts must include a data availability statement. This statement should provide the following information, where applicable:

- Accession codes, unique identifiers, or web links for publicly available datasets
- A description of any restrictions on data availability
- For clinical datasets or third party data, please ensure that the statement adheres to our policy

All unique materials such as patient-derived cell cultures are freely available and can be obtained by contacting the corresponding author with a standard MTA with Stanford University. Single cell and bulk RNAseq data in Extended Data Figure 1, Extended Data Figure 4 and Extended Data Figure 7 were analyzed from publicly available datasets on GEO (GSE102130, GSE134269, GSE94259 and GSE222560). Bulk RNASeq data used in Extended Data Figure 9 is available on GEO (GSE222481). Glioma Patch-Seq data referenced in the rebuttal is available on GEO (GSE222398). The hg38 and the hg19 reference genome was used for transcriptome annotations as stated in the methods. Data for all figures in the manuscript can be found in the Source Data, Supplementary Information and Supplementary Figure 1.

Field-specific reporting

Please select the one below that is the best fit for your research. If you are not sure, read the appropriate sections before making your selection.

☒ Life sciences ☐ Behavioural & social sciences ☐ Ecological, evolutionary & environmental sciences

For a reference copy of the document with all sections, see nature.com/documents/nr-reporting-summary-flat.pdf

Life sciences study design

All studies must disclose on these points even when the disclosure is negative.

Sample size	Based on the variance of xenograft growth in control mice, power calculations indicated use of at least 3 mice per treatment group/genotype to give 80% power to detect an effect size of 20% with a significance level of 0.05. For electrophysiological and calcium imaging studies, all studies were replicated across multiple cohorts of mice to verify reproducibility.
Data exclusions	No data were excluded from analyses.
Replication	All in vitro experiments have been performed in at least three independent coverslips for each experiment and performed in at least two independent experiments. The number of biological replicates (mice for in vivo growth, calcium imaging, electrophysiological recording, optogenetic stimulation and immuno-electron microscopy experiments) is indicated in the figure legends and was three or greater for all experiments and replicated in at least two independent experiments.
Randomization	All mice xenografted with tumor were randomized in assigning to treatment and control groups for all experiments.
Blinding	The experimenters performing histological quantifications were blinded to group allocation.

Reporting for specific materials, systems and methods

We require information from authors about some types of materials, experimental systems and methods used in many studies. Here, indicate whether each material, system or method listed is relevant to your study. If you are not sure if a list item applies to your research, read the appropriate section before selecting a response.

Materials & experimental systems

n/a	Involved in the study	
☐	☒	Antibodies
☐	☒	Eukaryotic cell lines
☒	☐	Palaeontology and archaeology
☐	☒	Animals and other organisms
☒	☐	Human research participants
☒	☐	Clinical data
☒	☐	Dual use research of concern

Methods

n/a	Involved in the study	
☒	☐	ChIP-seq
☒	☐	Flow cytometry
☒	☐	MRI-based neuroimaging

Antibodies

Antibodies used	Primary antibodies used in immunohistochemistry: chicken anti-GFP (1:500, #ab13970, Lot #GR3361051), mouse anti-Human Nuclei, clone 235-1 (1:200, MAB1281, Lot #3543073), guinea pig anti-Synapsin (1:500, #106-004 Synaptic Systems), rabbit anti-RFP (1:500, #600-401-379, Lot # 42872), mouse anti-nestin (1:500, #ab6320, Abcam) chicken anti-Neurofilament-H (1:1000, #NFH,

Lot#NFH957980), chicken anti-Neurofilament-M (1:1000, #NFM, Lot#NFM87837981), rabbit anti-microtubule-associated protein 2 (1:500, MAP2; EMD Millipore, #AB5622, LOT# 3897281), rabbit anti-Ki67 (1:500, ab15580, Abcam, LOT#GR3445754-1).
For secondary antibodies: Alexa 488 donkey anti-mouse IgG (#715-545-150), Alexa 594 donkey anti-rabbit IgG (#711-585-152), Alexa 647 donkey anti-mouse IgG (#715-605-150), Alexa 406 donkey anti-guinea pig IgG (#706-475-148) all used at 1:500 (Jackson Immuno Research).
For antibodies used in western blot: anti-GluA4 (#8070, Lot# 1), anti-GluA3 (#4676, Lot#1), anti-TrkB (#4606, Lot# 1), anti-GAPDH (#5174, Lot# 8), anti-beta-actin (#4970, Lot# 15), anti-phospho Erk (#4370, Lot# 17), anti-Erk (#9102, Lot# 26), anti-phospho Akt (#4060, Lot# 25), anti-Akt (#9272, Lot# 28), anti-phospho CAMKII (#12716, Lot# 4), anti-CAMKII (#4436, Lot# 3) all rabbit primary antibodies used at 1:1000 from Cell Signaling Technologies. Antibodies sourced from other companies were: rabbit anti_phospho-GluA4 (#PA5-36807, Invitrogen, Lot# SI2447842A) and rabbit anti-phospho TrkB (#SAB4503785, Lot# 110035).

Validation

All antibodies have been validated either in the literature, by manufacturers and/or in Antibodypedia for use in mouse immunohistochemistry and human western blot. To further validate the antibodies in our hands, we confirmed each antibody had either staining in the expected cellular patterns and brain-wide distributions for immunohistochemistry or for western blot, bands appearing at the correct weight according to the protein ladders run alongside the samples (LI-COR Chemiluminescent Protein Ladder (#NC0986471, Fisher Scientific) and Precision Plus Protein Dual Color Standards (#1610374, Bio-Rad).

Antibody validation information : Chicken anti-GFP (#ab13970, Reactivity : Species independent. Manufacturer validation: ICC: GFP-transfected NIH/3T3 Mouse embryo fibroblast cell line. Publication Figure : ExtData Fig1a and 6a, IF of human DIPG xenograft), mouse anti-Human Nuclei, clone 235-1 (MAB1281, Reactivity : human only, Manufacturer validation: IF neural stem cells transplanted into rat brain. Publication Figure : Fig1c, 1l, ExtData Fig5b, 5d, 6a, 10g, IF of human DIPG xenograft), guinea pig anti-Synapsin (#106-004, Reactivity : human, mouse, hamster, cow, zebrafish. Manufacturer validation: ICC immunostaining of PFA fixed rat hippocampus neurons. Publication Figure : 4e, IF of mouse neuron culture), rabbit anti-RFP (#600-401-379, Reactivity : RFP, Manufacturer validation: IF HopERCre/+; R26Tom/+ mice tissue. Publication Figure : 4e, IF of patient-derived PSD95-RFP+ DIPG culture), mouse anti-nestin (#ab6320, Reactivity : human, Manufacturer validation: IF human fetal neural progenitor cells, Publication Figure : Fig1i IF of human DIPG culture), chicken anti-Neurofilament-H (#NFH, Reactivity : human, mouse, rat, chicken, Manufacturer validation: IF rat cortical neurons and glia. Publication Figure : 4e, IF of mouse neuron culture), chicken anti-Neurofilament-M (#NFM, Reactivity : human, mouse, rat, bovine, chicken. Publication Figure : 4e, IF of mouse neuron culture), rabbit anti-microtubule-associated protein 2 (#AB5622, Reactivity : human, mouse, rat. Manufacturer validation: culture Rat hippocampal neurons. Publication Figure : 5b and 5d, IF of mouse neuron culture), rabbit anti-Ki67 (ab15580, Reactivity : mouse, human. Manufacturer validation: IF in Mef1 and HeLa cultures, Human skin tissue. Publication Figure : Fig1c, 1l, ExtData Fig 10g, IF of human DIPG xenograft). All primary antibodies used for IF in this publication have been used and validated in similar experimental paradigms previously (Venkatesh et al, Nature, 2019).

For antibodies used in western blot: anti-GluA4 (#8070, Reactivity : human, mouse, rat. Manufacturer validation: WB analysis of extracts from mouse brain, NIH/3T3 cells, rat brain, and C6 cells. Publication Figure : Fig3b, 3d, 3f and ExtData Fig. 6e, 6g WB analysis of human DIPG cell culture), anti-GluA3 (#4676, Reactivity : human, mouse, rat. Manufacturer validation: WB analysis of extracts from mouse and rat brains. Publication Figure : Fig3d, WB analysis of human DIPG cell culture), anti-TrkB (#4606, Reactivity : human. Manufacturer validation: Western blot analysis of extracts from NIH/3T3, NIH/3T3-TrkA, NIH/3T3-TrkB and NIH/3T3-TrkC cells. Publication Figure : ExtData Fig 1g, 2a, 10b, WB analysis of human DIPG cell culture), anti-GAPDH (#5174, Reactivity : human, mouse, rat, monkey. Manufacturer validation: Western blot analysis of extracts from HeLa, NIH/3T3, C6 and COS-7 cells. Publication Figure : Fig 3b, 3d, 3f , WB analysis of human DIPG cell culture), anti-beta-actin (#4970, Reactivity : human, mouse, rat, monkey, bovine, pig. Manufacturer validation: Western blot analysis of extracts from HeLa, NIH/3T3, C6 and COS-7 cells. Publication Figure : ExtData Fig 1g, 2a, 10b, 8a, 8e, 8g, 10b, WB analysis of human DIPG cell culture), anti-phospho Erk (#4370, Reactivity : human, mouse, rat, monkey. Manufacturer validation: Western blot analysis of extracts from NIH/3T3, C6 and COS-7 cells. Publication Figure : ExtData Fig : 2a, 8a, WB analysis of human DIPG cell culture), anti-Erk (#9102, Reactivity : human, mouse, rat. Manufacturer validation: Western blot analysis of extracts from HeLa cells. Publication Figure : ExtData Fig : 2a, 8a, WB analysis of human DIPG cell culture), anti-phospho Akt (#4060, Reactivity : human, mouse, rat. Manufacturer validation: Western blot analysis of extracts from PC-3 cells. Publication Figure : ExtData Fig : 8a, WB analysis of human DIPG cell culture), anti-Akt (#9272, Reactivity : human, mouse, rat. Manufacturer validation: Western blot analysis of extracts from CHO cells. Publication Figure : ExtData Fig : 8a, WB analysis of human DIPG cell culture), anti-phospho CAMKII (#12716, Reactivity : human, mouse, rat. Manufacturer validation: Western blot analysis of extracts from MKN-45 cells. Publication Figure : ExtData Fig : 8a, WB analysis of human DIPG cell culture), anti-CAMKII (#4436, Reactivity : human, mouse, rat. Manufacturer validation: Western blot analysis of extracts from 293T cells. Publication Figure : ExtData Fig : 8a, WB analysis of human DIPG cell culture), rabbit anti_phospho-GluA4 (#PA5-36807, Reactivity : human, mouse, rat. Manufacturer validation: Western blot analysis of extracts from HEK-293T cells. Publication Figure : ExtData Fig : 8e, 8g, WB analysis of human DIPG cell culture) and rabbit anti-phospho TrkB (#SAB4503785, Reactivity : human, mouse, rat. Manufacturer validation: Western blot analysis of extracts from NIH-3T3 cells. Publication Figure : ExtData Fig : 2a, 8a, WB analysis of human DIPG cell culture).

Eukaryotic cell lines

Policy information about cell lines

Cell line source(s)

The eukaryotic cell cultures used are patient-derived cultures of high-grade gliomas generated in the Monje lab from biopsy (SU-pcGBM2) or autopsy tissue (SU-DIPG-VI and SU-DIPGXIII-FL/P*), or provided by collaborators from a biopsy (QCTB-R059). 293T cells (ATCC) were used only for virus production.

Authentication

Short Tandem Repeat (STR) fingerprinting is performed every 3 months on all cell cultures to ensure authenticity.

Mycoplasma contamination

All cell cultures are routinely tested for mycoplasma contamination and all cultures were tested negative.

Commonly misidentified lines
(See ICLAC register)

No commonly misidentified lines were used.

Animals and other organisms

Policy information about studies involving animals; ARRIVE guidelines recommended for reporting animal research

Laboratory animals	All animal experiments were conducted in accordance with protocols approved by the Stanford University Institutional Animal Care and Use Committee (IACUC) and performed in accordance with institutional guidelines. Animals were housed according to standard guidelines with unlimited access to water and food, under a 12 hour light : 12 hour dark cycle, a temperature of 69.7F and 60% humidity. Both male and female NOD-SCID-IL2R gamma chain-deficient (NSG) were used between 1-7months of age. BDNF-TMKI mice were back-bred to NSG mice to facilitate orthotopic xenografting and used at 4-12 weeks of age.
Wild animals	No wild animals were used.
Field-collected samples	No field-collected samples were used.
Ethics oversight	All animal experiments were conducted in accordance with protocols approved by the Stanford University Institutional Animal Care and Use Committee (IACUC) and performed in accordance with institutional guidelines. The IACUC implements regulations from the United States Department of Agriculture (USDA), the Public Health Service (PHS) Policy, California State Regulations and Stanford University Polices and Guidlines to ensure effective and ethical animal research programs.

Note that full information on the approval of the study protocol must also be provided in the manuscript.
