# Supplementary material for: Glioma synapses recruit mechanisms of adaptive plasticity
Source: Nature. 2023 Nov 1;623(7986):366–74. doi: 10.1038/s41586-023-06678-1 (PMC10632140; doi:10.1038/s41586-023-06678-1)
Supplement: Supplementary file 4 — Additional statistical information. [file 41586_2023_6678_MOESM4_ESM.docx]

| **Panel** | **Statistical test** | **P value** | **Confidence interval** |
| --- | --- | --- | --- |
| Fig. 1d | One-way ANOVA with Tukey’s post hoc analysis | WT ChR2-ve vs WT ChR2+ve P = 0.0001 ;  WT ChR2+ve vs WT Bdnf-TMKI ChR2+ve P = 0.0003 | 95% CI  [-0.1416 to -0.04833] ;  [0.04456 to 0.1496] |
| Fig. 1e | Two-tailed log rank analyses | P = 0.001273 | 95% CI  [0.3208 to 2.440] |
| Fig. 1f | Two-tailed log rank analyses | SU-DIPGVI P = 0.013171 ;  SU-pcGBM2 P = 0.003203 | 95% CI  [0.2947 to 2.396] ;  [0.2848 to 2.315] |
| Fig. 1g | Two-tailed log rank analyses | P = 0.049986 | 95% CI  [0.3065 to 2.419] |
| Fig. 1h | One-way ANOVA with Tukey’s post hoc analysis | P = 0.0008 | 95% CI  [-0.1125 to -0.02993] |
| Fig. 1i | One-way ANOVA with Tukey’s post hoc analysis | WT monoculture vs WT coculture P = 0.0009 ;  WT vs NTRK2-KO coculture P = 0.0057 | 95% CI  [-0.5212 to -0.1753] ;  [0.08761 to 0.4335] |
| Fig. 1j | One-way ANOVA with Tukey’s post hoc analysis | WT vehicle vs WT NBQX P = 0.0016 ;  WT vehicle vs NTRK2-KO vehicle P = 0.0037 | 95% CI  [0.1164 to 0.3913]  [0.08537 to 0.3603] |
| Fig. 1m | One-way ANOVA with Tukey’s post hoc analysis | WT vehicle vs WT perampanel P = 0.0002 ; WT vehicle vs NTRK2-KO vehicle P = 0.0011 | 95% CI  [0.08206 to 0.2605]  [0.06013 to 0.2543] |
| Fig. 2c | Two-tailed paired Student’s *t*-test | P = 0.0082 | 95% CI  [17.70 to 89.72] |
| Fig. 2e | Two-tailed paired Student’s *t*-test | Control vs BDNF P = 0.0463 ;  Control KN-92 vs BDNF KN-92 P = 0.0416 | 95% CI  [1.477 to 122.2]  [3.115 to 108.1] |
| Fig. 2h | Two-tailed paired Student’s *t*-test | P = 0.0061 | 95% CI  [1.952 to 6.239] |
| Fig. 2k | Two-tailed paired Student’s *t*-test | P = 0.0001 | 95% CI  [0.7419 to 1.458] |
| Fig. 2l | Two-tailed paired Student’s *t*-test | P <0.0001 | 95% CI  [2.082 to 3.363] |
| Fig. 3c | Two-tailed unpaired Student’s *t*-test | Control vs BDNF 5min  P = 0.0046 ;  Control vs BDNF 15min  P = 0.0004 ;  Control vs BDNF 30min  P = 0.0020 | 95% CI  [14.43 to 41.63] ;  [49.73 to 84.99] ;  [27.32 to 62.17] |
| Fig. 3e | Two-tailed unpaired Student’s *t*-test | P = 0.0189 | 95% CI  [6.886 to 43.84] |
| Fig. 3j | Two-tailed unpaired Student’s *t*-test | pHluorin pH 7.4 vs pH 5.5  P = 0.0077 ;  pH 5.5 vs pH 7.4 P = 0.0110 | 95% CI  [-1.987 to -0.6668]  [0.6481 to 2.321] |
| Fig. 3l | Two-tailed one-sample *t*-test | pHluorin 0min vs 5min  P = 0.0462 ;  0min vs 15min P = 0.0045 ; 0min vs 20min P = 0.0037 | 95% CI  [0.01108 to 0.9886]  [0.2965 to 1.100]  [0.2045 to 0.7134] |
| Fig. 3m | Two-tailed unpaired Student’s *t*-test | P = 0.0248 | 95% CI  0.08878 to 1.090 |
| Fig. 4b | Two-sided Fisher’s exact test | P <0.0001 | 95% CI  [1.452 to 2.139] |
| Fig. 4d | Two-tailed unpaired Mann-Whitney test | P = 0.0012 | 96.50% CI of difference  [-9.137 to -2.614] |
| Fig. 4f | Two-tailed unpaired Student’s *t*-test | P <0.0001 | 95% CI  [-27.31 to -10.54] |
| Fig. 4g | Two-tailed unpaired Student’s *t*-test | P = 0.0132 | 95% CI  [-24.84 to -3.124] |
| Fig. 4i | Two-tailed one-sample *t*-test | Control vs 5ms P = 0.0410; Control vs 25ms P = 0.0046 | 95% CI  [3642 to 105836]  [67059 to 193947] |

**Supplementary Table 1: Additional Statistical Information**. WT, wild-type; KO, knockout; CI, confidence interval
